# Polymorphisms -455G/A and -148C/T and Fibrinogen Plasmatic Level as Risk Markers of Coronary Disease and Major Adverse Cardiovascular Events

**DOI:** 10.1155/2019/5769514

**Published:** 2019-07-01

**Authors:** Luis Miguel Canseco-Avila, Alexander Lopez-Roblero, Eleazar Serrano-Guzman, Javier Aguilar-Fuentes, Carlos Jerjes-Sanchez, Augusto Rojas-Martinez, Rocio Ortiz-Lopez

**Affiliations:** ^1^Laboratorio de Investigación, Hospital Regional de Alta Especialidad Ciudad Salud, Carretera Tapachula-Puerto Madero Km 15 Sin Número Colonia los Toros, Tapachula, Chiapas, Mexico; ^2^Facultad de Ciencias Químicas, Campus IV, Universidad Autónoma de Chiapas, Tapachula, Chiapas, Mexico; ^3^Facultad de Ciencias Agrícolas, Universidad Autónoma de Chiapas, Huehuetán, Chiapas, Mexico; ^4^Tecnologico de Monterrey, Escuela de Medicina y Ciencias de la Salud, Monterrey, Mexico

## Abstract

Some polymorphisms in genes codifying for fibrinogen have been correlated with plasma levels of this protein, and several studies reported their associations with acute cardiovascular events. In the present study, 118 subjects with unstable and stable coronary diseases were enrolled to determinate the associations among fibrinogen gene polymorphisms, plasma fibrinogen levels, and major cardiovascular adverse events in a sample of southwestern Mexico. The groups, including 81 control subjects, were matched for age, sex, body mass index, and sedentarism. Plasma fibrinogen levels and the polymorphisms 455G/A, -148C/T, +1689T/G, and *Bcl* I of the gene of fibrinogen were compared in all groups. Plasma fibrinogen levels (>465 mg/dl) were significant in patients with coronary disease. Fibrinogen plasma values > 450 mg/dl were associated with cardiovascular mortality during the follow-up analysis of the unstable coronary disease group (*p* = 0.04). The allelic loads of -455A and -148T were associated with plasma fibrinogen levels > 450 mg/dl (*p* < 0.003 and *p* = 0.03, respectively) and with coronary disease (*p* = 0.016 and *p* < 0.006, respectively). The follow-up of posterior events after an acute coronary event showed that the genetic load of the -148T allele was associated with major adverse cardiovascular events (RR = 1.8, 95%CI = 1.01‐3.35, *p* = 0.04). Fibrinogen plasmatic levels > 450 mg/dl and the fibrinogen polymorphisms -455G/A and 148C/T had association with MACE and coronary disease. This study suggests that these gene polymorphisms are associated with cardiovascular risk.

## 1. Introduction

The role of fibrinogen gene polymorphisms and protein levels as inflammation markers of cardiovascular risk has been evaluated in several epidemiological studies excluding Latin-American populations [1–4]. *In vitro* studies suggest that the synthesis of the *β* chain of fibrinogen is the limiting factor for the production of fibrinogen. Some polymorphisms of this gene may be associated with elevated levels of plasma fibrinogen. Since studies associating fibrinogen polymorphisms and cardiovascular risk have not included Latin-American populations [5, 6], we studied the associations among fibrinogen polymorphisms, plasma levels of the protein, and coronary disease, including unstable coronary disease (UCD) and stable coronary disease (SCD), and their associations with major adverse cardiovascular events (MACE) in a sample of southwestern Mexico.

## 2. Materials and Methods

### 2.1. Study Design

The study design is prospective and controlled, with a six-month follow-up study. Inclusion criteria are (a) age between 35 and 75 years, (b) UCD and SCD with secondary atherothrombosis, and (c) clinical stability. Exclusion criteria are (a) age greater than 75 years; (b) UCD secondary effort stress, uncontrolled hypertension, aortic stenosis, acute or chronic pulmonary arterial hypertension, thyrotoxicosis, anemia, heart failure, excessive vasodilatation, etc.; (c) Killip class III or IV; (d) acute coronary event in the last three months; (e) ejection fraction < 35%; (f) SCD without atherothrombosis; (g) hematological, hepatic, or neoplastic disease; (h) acute or chronic inflammatory activity; and (i) excessive alcohol consumption. The control group is composed of apparently healthy subjects. All groups were matched for age, sex, body mass index, and sedentarism, and all samples were obtained under similar conditions. This study was conducted in accordance with the Declaration of Helsinki principles and was approved by the Ethics Committee of the Hospital Regional de Alta Especialidad “Ciudad Salud” in Tapachula, Mexico. All subjects signed an informed consent before entering the study.

### 2.2. Blood Sample and Laboratory Analysis

Because of the exquisite sensitivity of the plasma markers in *in vitro* manipulations, the samples were obtained with extreme care. Once the patient signed the informed consent, two people with special training did a venous puncture with a vacuum test tube. 3 milliliters of venous blood was collected in a tube with sodium citrate (ACD) and another 10 milliliters in a tube with EDTA. The puncture was done with a 20-gauge needle in a region of the arm with no cracks, folds, or skin breaks. The sample was always obtained before starting any fibrinolytic, antithrombotic, and antiplatelet treatments or invasive procedures, including placement of intravenous lines. The anticoagulated samples with ACD were centrifuged immediately at a temperature of 4°C, and the plasma obtained was used to determine the levels of plasma fibrinogen by the von Clauss method [7]. The levels of plasma fibrinogen were divided into three groups: low (<350 mg/dl), intermediate (350 to 450 mg/dl), and high (>450 mg/dl). The anticoagulated material with EDTA was used for the isolation of DNA. Laboratory personnel were blinded to the clinical diagnosis of the subjects.

### 2.3. Analysis of Polymorphisms

DNA was extracted by standard procedures. Four polymorphisms of the fibrinogen B gene were analyzed, two of them located in the promoter (-455G/A, -148C/T) and two located in the gene (Bcl-1 and +1689T/G), by means of the polymerase chain reaction (PCR) followed by splicing with restriction enzymes (PCR-RFLP). PCR primers were the following: for G/A^−455^ (HaeIII) and C/T^−148^ (HindIII/Alu) *F-5*′AAG AAT TTG GGA ATG CAA TCT CTG CTA CCT*-3*′ and *R-5*′CTC CTC ATT GTC GTT GAC ACC TTG GGA C*-3*′; for T/G^+1689^ (AvaII) *F-5*′TGG TTA ATC TGG TTA ACT CTG G*-3*′ and *R-5*′GTC ACT AGC TAT ACA TCC TTT G-3′; and for BclI^B1/B2^ F*-5*′ACC TGG TTT CTC TGC CAC AAG-3′ and R*-5*′AAT ACT TCT CAT ACC ACA GTG T-3′ [8]. Initiators were acquired from Invitrogen (Carlsbad, CA, USA) and the restriction enzymes from New England Biolabs (Ipswich, MA, USA).

PCR was carried out in volumes of 25 *μ*l containing 100 ng of DNA, 1x reaction buffer, 1.5 *μ*M of MgCl_2_, 0.5 *μ*M of dNTPs, 0.5 *μ*M of Taq polymerase, and 0.5 *μ*M of each initiator. The alignment temperatures varied from 55 to 61°C. The amplified and digested products were analyzed in 2 and 4% agarose gels, respectively.

In order to assure the genotyping quality, the results were validated in a posterior screening in 10% of randomly selected samples and all those with a homozygotic pattern for the mutant alleles, as proposed by Bladbjerg et al. [9]. Two independent observers analyzed the genotypes, and an additional observer solved any discrepancy.

### 2.4. Definitions

The following data are the definitions of terms used in the study:

1. Unstable coronary disease (UCD): acute ischemic chest pain > 20 minutes at rest, with ST (elevation or depression) and with or without necrosis
2. Stable coronary disease (SCD): previous ischemic heart disease secondary to atherosclerosis probed by coronary angiography and without history of any coronary acute event in the last one year
3. Healthy subject: participant without history of cardiovascular or chronic metabolic disease. ECG and laboratory test were performed in all participating subjects
4. Clinical stability: patients with coronary disease with normal blood pressure and without clinical manifestations of left ventricular dysfunction
5. Major adverse cardiovascular events (MACE): recurrent ischemia: new episode of chest pain > 5 minutes with ST abnormalities with or without enzymatic elevation
6. Reinfarction: acute ischemic chest pain > 20 minutes at rest, with ST (elevation or depression) and with new enzymatic elevation
7. Cardiogenic shock: systolic blood pressure < 100 mmHg, renal output < 20 ml/minute, echocardiographic ejection fraction < 40%, and clinical manifestations of left ventricular dysfunction
8. Cardiovascular mortality: death, secondary ventricular fibrillation or arrhythmia, infarction, acute ischemic event, and cardiogenic shock
9. Follow-up: all patients had six months of follow-up through office visit or telephone contact every three months

### 2.5. Statistical Analysis

The difference between the averages of the groups and the continuous variables was evaluated using a two-tailed Student *t*-test, evaluating the result by the Wilcoxon test. The discreet variables were analyzed by Chi-square using the Yates correction. A two-way analysis of variance (ANOVA) for repeated measures was performed to analyze differences in levels of fibrinogen between UCD, SCD, and healthy subjects. Logistic and multiple regression analysis models were used to assess the effect of fibrinogen plasma levels and its polymorphisms on MACE in the acute phase and in the follow-up. Survival Kaplan-Meier curves were considered, and odds ratio (OR) and relative risk (RR) with confidence intervals (CI) of 95% were also considered. Statistical significance was established for values of *p* < 0.05. The data were reported in percentages, averages, standard deviations, CI, and OR. All the analyses were done using a commercial statistical package (GB-STAT version 10.0 of Dynamic Microsystems Inc., Copyright 2004).

## 3. Results

### 3.1. The Characteristics of Patients and Controls

**Figure 1:**
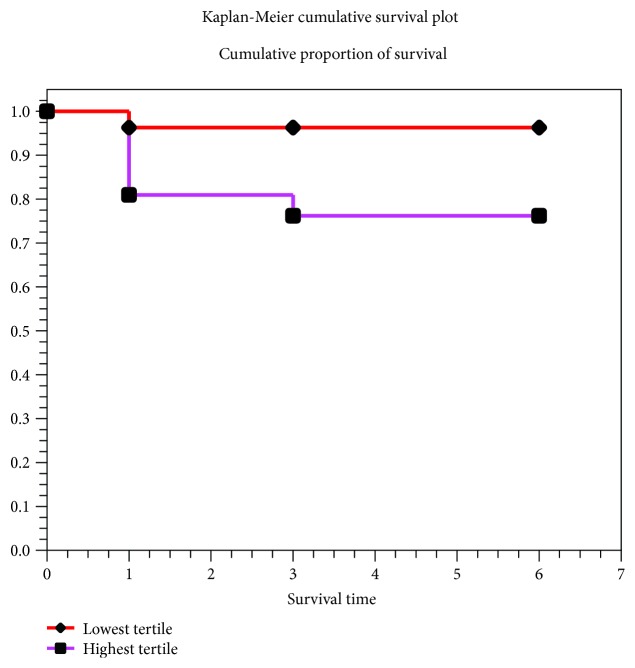
Highest tertile fibrinogen versus the lowest tertile in mortality to one year (*p* = 0.04).

From January 2015 to December 2017, 199 subjects were included in the study. Fifty patients with UCD and 68 with SCD, respectively, were admitted to the Hospital Regional de Alta Especialidad “Ciudad Salud,” for a total of 118 patients and 81 subjects in the control group. Demographic characteristics of the groups are shown in Table 1. No intergroup differences in age and gender were observed. The UCD group had more history of a previous acute coronary event, diabetes, hypertension, smoking, and lipid abnormalities. Over 50% in all groups did not have physical activity, and obesity was observed in all of them. Fibrinogen levels were significantly higher in patients with coronary disease in general with respect to controls (*p* = 0.001). In the six-month follow-up of UCD patients, high plasmatic fibrinogen levels correlated with cardiovascular mortality (*p* = 0.04) (Figure 1).

### 3.2. Genotype and Allele Frequency Distribution

Table 2 shows the distribution of genotypes and alleles in the three analyzed groups. All analyzed fibrinogen gene polymorphisms were in Hardy-Weinberg equilibrium (*p* > 0.05). Of the four studied polymorphisms, only those located in the fibrinogen B gene promoter (455G/A and -148C/T) were different between UCD, SCD, and controls. The frequency of the genotypes -455G/A and -148C/T was significantly more represented in the patients with coronary disease (*p* = 0.016 and *p* < 0.006, respectively). Nevertheless, when the analysis focused on allele frequencies, the variants -455 and -148T were significantly more frequent in patients with UCD and SCD when compared with controls (*p* = 0.01 and *p* = 0.0005, respectively). The rest of the alleles did not show significant values for a particular clinical condition.

The determinations of genetic loads of an individual allele among the possible studied genotypes demonstrated an increased OR for coronary disease. The load of the -455A allele was associated with SCD (OR = 3.35, 95%CI = 1.6‐7.1, *p* = 0.002) and UCD (OR = 3.19, 95%CI = 1.4‐7.2, *p* = 0.005), and an association was also observed for the load of the -148T allele with SCD (OR = 2.21, 95%CI = 1.1‐4.3, *p* = 0.02) and UCD (OR = 3.10, 95%CI = 1.5‐6.5, *p* = 0.003) (Table 3).

### 3.3. Fibrinogen Levels according to Genotype

The effect of the genotypes on plasma fibrinogen levels is shown in Table 4. Levels > 450 mg/dl of fibrinogen were associated with the polymorphisms -455G/A, -148C/T, +1689T/G, and Bcl-1. The genotypes -455G/A and +1689 were associated with high plasma levels of the protein (*p* < 0.003 and *p* = 0.02).

### 3.4. Multiple Regression Models

A multiple regression analysis determined that the genetic load of the -455A and -148T alleles is an independent predictor of elevated levels of fibrinogen (*r* = 0.3, *p* = 0.008), after considering other risk factors such as age, sex, BMI, dyslipidemia, diabetes, smoking, and sedentarism.

### 3.5. Follow-Up and Major Cardiovascular Adverse Events

The load of the T allele of the -148 variant was a predictor of MACE in the acute phase and recurrent ischemia in the one-year follow-up in the UCD group (Table 5). A logistic multiple regression analysis determined that the -148T allele is an independent predictor of in-hospital recurrent ischemia (RR = 1.8, 95%CI = 1.01‐3.35, *p* = 0.04) and MACE at the one-year follow-up (RR = 1.9, 95%CI = 1.07‐3.52, *p* = 0.02). Several modeling of the -455G/A, Bcl-1, Taq I, and +1689G/T polymorphisms showed poor or null correlations with plasma fibrinogen levels. The Kaplan-Meier survival analysis suggests a reduced survival rate in patients with UCD and highest plasma fibrinogen levels compared to those with the lowest levels (Figure 1).

## 4. Discussion

These results provide reliable information about baseline fibrinogen levels and gene polymorphisms and cardiovascular events in acute phases and in the 1-year follow-up in Mexican patients. The elevated fibrinogen levels correlated with coronary disease, as has been reported [2–4]. This study provides important insights about genetic and inflammatory risk profiles in UCD and SCD in Mexico and probably represents the first study in Latin-America [5].

Notably, the average plasma levels of this protein in subjects with coronary disease (465 mg/dl) and in controls (416 mg/dl) are greater than those reported in other populations using the von Clauss method to measure fibrinogen [10–12]. Although they are within reference values [13, 14], nevertheless, other evidence reports establish similar values [15, 16]. In any case, the differences observed among controls, UCD, and SCD suggest phenotypic stratifications that may be associated with an increased cardiovascular risk.

Another observation was that fibrinogen levels were predictors of cardiovascular mortality during the six-month follow-up in the UCD group. The values observed in UCD subjects who died during the study (468.3 ± 185.8 mg/dl) are similar to those reported in Israeli [1], Japanese-American [3], American [17], and Spanish populations [18].

Analysis of the allelic frequencies of the studied polymorphisms shows that only the -455A and -148T alleles are associated with coronary disease in general (UCD and SCD together), whereas the study of genotypes indicates that the genotypes -455G/A and -148 C/T and T/T are associated with coronary disease. The association between coronary disease and the -455 polymorphism has been reported in the majority of the ethnic groups around the world [5, 19–23]; however, other studies did not find this association [4, 24]. Although the evidence on the -148 variant as a cardiovascular risk marker is scarce, it has been associated with myocardial infarction in an Asian population [22] and has been considered as a predictor of carotid atherosclerosis [25]. The alleles +1689G and Bcl-1 B2 did not show associations with cardiovascular risk, as has been reported [4].

The genetic loads of the -455A and -148T alleles conferred relevant ORs for SCD and UCD, corroborating their clinical effect. These polymorphisms were associated with fibrinogen levels > 450 mg/dl, and the genotype -455G/A was the most significant (*p* < 0.003) followed by +1689G/T, -148C/T, and Bcl-1 (*p* = 0.02, *p* = 0.03, and *p* = 0.03, respectively). It is important to emphasize that these results derive from a comparison between polymorphisms and fibrinogen levels without discriminating particular clinical conditions.

The most relevant finding of this work is the almost twice greater risk of recurrent ischemia and MACE in the 1-year follow-up in UCD patients carrying the -148T allele. Our study shows that the -148T allele is highly associated with increased levels of plasmatic fibrinogen, coronary disease, and adverse events after a UCD event. In addition, the -455A allele is associated with high values of plasmatic fibrinogen and coronary disease. The simultaneous association of the alleles -455A and -148T agrees with the hypothesis of a genetic linkage, as reported in Caucasian populations [26, 27].

The two gene polymorphisms relevant for this study are located in the promoter region of the fibrinogen B gene, very close to the elements of response to IL-6 and C/EBP and of the HNF1 and HNF3 [4, 5, 28–31]. They may modify the interaction of the promoter with IL-6, a cytosine responsible for the inflammatory response in the acute phase of a coronary event, and this explains the associations observed in this study.

## 5. Conclusions

Plasmatic fibrinogen levels > 450 mg/dl and the polymorphisms -455G/A and 148C/T of the fibrinogen gene are association with MACE and coronary disease in a sample of a Mexican population.

## Figures and Tables

**Table 1:** Basal characteristics of the study population.

Characteristic	Controls^a^ *N* = 81 (%)	SCD^b^ *N* = 68 (%)	UCD^c^ *N* = 50 (%)	*p* value
Age (yr, ±SD)	59.3 ± 9.5	59.5 ± 10	60.5 ± 8.4	NS
Female	29 (35.8)	23 (33.8)	17 (34)	NS
Male	52 (64.2)	45 (66.2)	33 (66)	
*Familiar antecedents*				
Diabetes	26 (32.1)	26 (38.2)	27 (50)	0.043
CD	13 (16.0)	35 (51.5)	15 (30)	<0.00001
Hypertension	23 (28.4)	30 (44.1)	23 (46)	NS
*Risk factors*				
Diabetes	16 (19.8)	38 (55.9)	27 (54)	<0.00001
Hypertension	27 (33.3)	42 (61.8)	30 (60)	0.00062
Smoking	23 (28.4)	31 (45.6)	27 (54)	0.00901
Dyslipidemia	3 (3.7)	14 (20.6)	13 (26)	<0.00010
Sedentary life	44 (54.3)	37 (54.4)	29 (58)	NS
BMI	27.8 ± 4.2	26.3 ± 3.4	29.7 ± 3.1	NS
*Index event*				
ST elevation	—	—	29 (58)	
Non-ST elevation	—	—	21 (42)	
*Biochemical variable*				
Fibrinogen (mg/dl)	416.3 ± 60.4	465.5 ± 107.9	547.1 ± 196.2	0.001

NS = not significant (*p* > 0.05).

**Table 2:** Fibrinogen genotypes and alleles.

Genotype	Controls *N* = 81 (%)	SCD *N* = 68 (%)	UCD *N* = 50 (%)	*p* value
-455 Fg				**0.016** ^∗^
G/G	67 (82.7)	40 (58.8)	30 (60)	
G/A	12 (14.8)	26 (38.2)	20 (40)	
A/A	2 (2.5)	2 (3.0)	0 (0)	
+1689 Fg				0.932^∗^
T/T	55 (67.9)	44 (64.7)	30 (60)	
G/T	25 (30.9)	23 (33.8)	19 (38)	
G/G	1 (1.2)	1 (1.5)	1 (2)	
Bcl-1 Fg				0.999^∗^
B1/B1	62 (76.5)	53 (78)	39 (78)	
B1/B2	19 (23.5)	15 (22)	11 (22)	
B2/B2	0 (0)	0 (0)	0 (0)	
-148 Fg				**0.006** ^∗^
C/C	48 (59.3)	27 (39.7)	16 (32)	
C/T	31 (38.3)	34 (50)	24 (48)	
T/T	2 (2.4)	7 (10.3)	10 (20)	
*Alleles*				
-455				
G	146 (0.9012)	106 (0.7794)	80 (0.8000)	**0.0100**
A	16 (0.0988)	30 (0.2206)	20 (0.2000)	
+1689				
T	135 (0.8333)	111 (0.8162)	79 (0.7900)	0.4350
G	27 (0.1667)	19 (0.1838)	21 (0.2100)	
Bcl-1				
B1	143 (0.8827)	121 (0.8897)	89 (0.8900)	0.9760
B2	19 (0.1173)	15 (0.1103)	11 (0.1100)	
-148				
C	127 (0.7840)	88 (0.6471)	56 (0.5600)	**0.0005**
T	35 (0.2160)	48 (0.3529)	44 (0.4400)	

SCD: stable coronary disease; UCD: unstable coronary disease; Fg: fibrinogen. ^∗^Yates' *p* value.

**Table 3:** Allelic load of the alleles -455A and -148T.

Genotype carriers	Controls^a^ *N* = 81 (%)	SCD^b^ *N* = 68 (%)	UCD^c^ *N* = 50 (%)	OR (95% CI), *p*
-455 Fg				
G/G	67	40	30	3.35 (1.6-7.1), 0.002^ab^
G/A+A/A	14	28	20	3.19 (1.4-7.2), 0.005^ac^
				0.95 (0.4-2.0), 0.898^bc^
-148 Fg				
C/C	48	27	16	2.21 (1.1-4.3), 0.020^ab^
C/T+T/T	33	41	34	3.10 (1.5-6.5), 0.003^ac^
				1.40 (0.6-3.0), 0.391^bc^

ab = controls versus SCD; ac = controls versus UCD; bc = SCD versus UCD.

**Table 4:** Relationship between plasma fibrinogen levels and polymorphisms.

Fibrinogen *n* = 199	<350 mg/dl *n* = 58	350-450 mg/dl *n* = 81	>450 mg/dl *n* = 60	*p* value
Bcl-1 Fg				
B1/B1	51 (88)	59 (73)	40 (67)	**0.03**
B1/B2	6 (10)	20 (24)	20 (33)	
B2/B2	1 (2)	2 (3)	0 (0)	
-455 Fg				**0.003**
G/G	44 (76)	55 (68)	30 (50)	
G/A	12 (21)	26 (32)	30 (50)	
A/A	2 (3)	0 (0)	0 (0)	
-148 Fg				**0.03**
C/C	30 (52)	38 (47)	22 (36)	
C/T	20 (34)	40 (49)	27 (43)	
T/T	8 (14)	3 (4)	11 (21)	
+1689Fg				**0.02**
T/T	42 (72)	55 (68)	29 (48)	
G/T	15 (26)	26 (32)	31 (52)	
G/G	1 (2)	0 (0)	0 (0)	

**Table 5:** Adverse cardiovascular events to one year.

UCD (*n* = 50) polymorphism	Nonevent (*n* = 17)	Adverse event (*n* = 33)	Type of MACE (one year)			
			Angina (*n* = 30)	Reinfarct (*n* = 5)	Shock (*n* = 5)	Death (*n* = 7)
-455						
G/G	9	19	16	3	4	4
G/A+A/A	8	14	14	2	1	3
-148						
C/C	10	8	6	0	2	3
C/T+T/T	7	25^∗^	24^**#**^	5	3	4
Bcl-1						
B1/B1	13	26	22	4	3	4
B1/B2+B2/B2	4	7	8	1	2	3
+1689						
T/T	9	19	17	3	5	4
G/T+G/G	8	14	13	2	0	1

^∗^RR = 1.8, 95%CI = 1.01‐3.35, *p* = 0.04; ^**#**^RR = 1.9, 95%CI = 1.07‐3.52, *p* = 0.02.

## Data Availability

The data used to support the findings of this study are available from the corresponding author upon request.

## References

[1] Benderly M., Graff E., Reicher-Reiss H., Behar S., Brunner D., Goldbourt U. (1996). Fibrinogen is a predictor of mortality in coronary heart disease patients. *Arteriosclerosis, Thrombosis, and Vascular Biology*.

[2] Yarnella J. W. G., Patterson C. C., Sweetnamb P. M., Lowec G. D. O. (2004). Haemostatic/inflammatory markers predict 10-year risk of IHD at least as well as lipids: the Caerphilly collaborative studies. *European Heart Journal*.

[3] Yano K., Grove J. S., Chen R., Rodriguez B. L., Curb J. D., Tracy R. P. (2001). Plasma fibrinogen as a predictor of total and cause-specific mortality in elderly Japanese-American men. *Arteriosclerosis, Thrombosis, and Vascular Biology*.

[4] Doggen C. J. M., Bertina R. M., Cats V. M., Rosendaal F. R. (2000). Fibrinogen polymorphisms are not associated with the risk of myocardial infarction. *British Journal of Haematology*.

[5] Iacoviello L., Vischetti M., Zito F., Benedetta Donati M. (2001). Genes encoding fibrinogen and cardiovascular risk. *Hypertension*.

[6] Humphries S. E., Henry J. A., Montgomery H. E. (1999). Gene-environment interaction in the determination of levels of haemostatic variables involved in thrombosis and fibrinolysis. *Blood Coagulation & Fibrinolysis*.

[7] Clauss A. (1957). Rapid physiological coagulation method in determination of fibrinogen. *Acta Haematologica*.

[8] Thomas A., Lamlum H., Humphries S., Green F. (1994). Linkage disequilibrium across the fibrinogen locus as shown by five genetic polymorphisms, G/A ^−455^ (*Hae*III), C/T ^−148^ (*Hind*III/*Alu*I), T/G^+1689^ (*Ava*II), and *Bcl*I (*β*‐fibrinogen) and *Taq*I (*α*‐fibrinogen), and their detection by PCR. *Human Mutation*.

[9] Bladbjerg E.-M., Gram J., Jespersen J., de Maat M. P. M. (2002). Internal quality control of PCR-based genotyping methods: practical experiences. *Vascular Pharmacology*.

[10] Heinrich J., Balleisen L., Schulte H., Assmann G., van de Loo J. (1994). Fibrinogen and factor VII in the prediction of coronary risk. Results from the PROCAM study in healthy men. *Arteriosclerosis and Thrombosis: A Journal of Vascular Biology*.

[11] Wilhelmsen L., Svardsudd K., Korsan-Bengtsen K., Larsson B., Welin L., Tibblin G. (1984). Fibrinogen as a risk factor for stroke and myocardial infarction. *The New England Journal of Medicine*.

[12] Stec J. J., Silbershatz H., Tofler G. H. (2000). Association of fibrinogen with cardiovascular risk factors and cardiovascular disease in the Framingham Offspring Population. *Circulation*.

[13] Koenig W. (2003). Fibrin(ogen) in cardiovascular disease: an update. *Thrombosis and Haemostasis*.

[14] Kamath S., Lip G. Y. H. (2003). Fibrinogen: biochemistry, epidemiology and determinants. *QJM*.

[15] Yarnell J. W., Baker I. A., Sweetnam P. M. (1991). Fibrinogen, viscosity, and white blood cell count are major risk factors for ischemic heart disease. The Caerphilly and Speedwell collaborative heart disease studies. *Circulation*.

[16] Cremer P., Nagel D., Seidel D., van de Loo J. C., Kienast J. (1996). Considerations about plasma fibrinogen concentration and the cardiovascular risk: combined evidence from the GRIPS and ECAT studies. Goettingen Risk Incidence and Prevalence Study. European Concerted Action on Thrombosis and Disabilities. *The American Journal of Cardiology*.

[17] Acevedo M., Pearce G. L., Kottke-Marchant K., Sprecher D. L. (2002). Elevated fibrinogen and homocysteine levels enhance the risk of mortality in patients from a high-risk preventive cardiology clinic. *Arteriosclerosis, Thrombosis, and Vascular Biology*.

[18] Arnau Vives M. A., Rueda Soriano J., Martínez Dolz L. V. (2002). Prognostic value of fibrinogen in patients admitted with suspected unstable angina and non-q-wave myocardial infarction. *Revista Española de Cardiología*.

[19] Toss H., Lindahl B., Siegbahn A., Wallentin L., for the FRISC Study Group (1997). Prognostic influence of increased fibrinogen and C-reactive protein levels in unstable coronary artery disease. *Circulation*.

[20] Pegoraro R. J., Ranjith N., Rom L. (2005). Coagulation gene polymorphisms as risk factors for myocardial infarction in young Indian Asians. *Cardiovascular Journal of South Africa*.

[21] de Maat M. P. M., Kastelein J. J. P., Jukema J. W. (1998). −455G/A polymorphism of the *β*-fibrinogen gene is associated with the progression of coronary atherosclerosis in symptomatic men: proposed role for an acute-phase reaction pattern of fibrinogen. *Arteriosclerosis, Thrombosis, and Vascular Biology*.

[22] Lam K. S. L., Ma O. C. K., Wat N. M. S., Chan L. C., Janus E. D. (1999). *β*-Fibrinogen gene G/A-455 polymorphism in relation to fibrinogen concentrations and ischaemic heart disease in Chinese patients with type II diabetes. *Diabetologia*.

[23] Carter A. M., Mansfield M. W., Stickland M. H., Grant P. J. (1996). *β*-Fibrinogen gene −455 G/A polymorphism and fibrinogen levels: risk factors for coronary artery disease in subjects with NIDDM. *Diabetes Care*.

[24] Tybjaerg-Hansen A., Agerholm-Larsen B., Humphries S. E., Abildgaard S., Schnohr P., Nordestgaard B. G. (1997). A common mutation (G-455--> A) in the beta-fibrinogen promoter is an independent predictor of plasma fibrinogen, but not of ischemic heart disease. A study of 9,127 individuals based on the Copenhagen City Heart Study. *The Journal of Clinical Investigation*.

[25] Schmidt H., Schmidt R., Niederkorn K. (1998). *β*-Fibrinogen gene polymorphism (C_148_→T) is associated with carotid atherosclerosis: results of the Austrian Stroke Prevention Study. *Arteriosclerosis, Thrombosis, and Vascular Biology*.

[26] Laffan M. A. (2001). Fibrinogen polymorphisms and disease. *European Heart Journal*.

[27] van ’t Hooft F. M., von Bahr S. J. F., Silveira A., Iliadou A., Eriksson P., Hamsten A. (1999). Two common, functional polymorphisms in the promoter region of the *β*-fibrinogen gene contribute to regulation of plasma fibrinogen concentration. *Arteriosclerosis, Thrombosis, and Vascular Biology*.

[28] Verschuur M., de Jong M., Felida L., de Maat M. P. M., Vos H. L. (2005). A hepatocyte nuclear factor-3 site in the fibrinogen *β* promoter is important for interleukin 6-induced expression, and Its activity is influenced by the adjacent –148C/T polymorphism. *The Journal of Biological Chemistry*.

[29] Anderson G. M., Shaw A. R., Shafer J. A. (1993). Functional characterization of promoter elements involved in regulation of human B beta-fibrinogen expression. Evidence for binding of novel activator and repressor proteins. *The Journal of Biological Chemistry*.

[30] Dalmon J., Laurent M., Courtois G. (1993). The human beta fibrinogen promoter contains a hepatocyte nuclear factor 1-dependent interleukin-6-responsive element. *Molecular and Cellular Biology*.

[31] Gervois P., Vu-Dac N., Kleemann R. (2001). Negative regulation of human fibrinogen gene expression by peroxisome proliferator-activated receptor *α* agonists via inhibition of CCAAT box/enhancer-binding protein *β*. *The Journal of Biological Chemistry*.

